# Learning Needs Assessment of Multidisciplinary Staff Working Within the Memory Assessment Services – Survey Study

**DOI:** 10.1192/bjo.2022.150

**Published:** 2022-06-20

**Authors:** Hafeesa Sameem, Raja Adnan Ahmed

**Affiliations:** Aneurin Bevan University Health Board, Newport, United Kingdom

## Abstract

**Aims:**

To assess the learning needs of the memory team multidisciplinary (MDT) staff working in South East Wales, in relation to diagnosis of dementia, types of dementia, anti-dementia medications and risk associated with dementia.

**Methods:**

An online anonymised survey was designed and distributed via email among memory team MDT staff of 3 different health boards of the South East Wales area which included Aneurin Bevan University Health Board, Cwm Taf Morgannwg University Health Board and Cardiff and Vale University Health Board.

The survey was designed to gather information about their understanding of the common type of dementia, treatment with medication, indications and cautions related to medications, the potential side effects and risks associated with Dementia. The survey had a mixture of `Likert Scale` format with free text segments where their opinion was sought in their own words.

**Results:**

Twenty-one (21) staff members responded to the survey. The majority (16) were psychiatric nurses, rest were psychologists and support workers.

Thirteen (13) staff members reported they feel confident in identifying subtypes of dementia whereas the rest reported they can benefit from additional knowledge. Majority of staff felt they understood the indications of anti-dementia medication side effects of medications and risks associated with Dementia but do not fully understand the contra-indications and cautions related to anti-dementia medications.

Participants suggested that regular teaching, lectures and updates should be arranged especially targeting the diagnostic criteria, medication and risk assessment. Sessions providing bite-size information on a regular basis to build on their knowledge base were suggested.

Participants also suggested shadowing doctors and consultants in memory clinics as a useful tool to improve their knowledge base.

**Conclusion:**

MDT staff working with memory teams are at the front line of screening, assessments and providing help and treatment to patients and their families. It is important to make sure our staff are equipped with evidence-based accurate knowledge and training.

This survey study indicated that MDT staff working within the memory team appreciate more training and helped identify the specific areas and mode of teaching required.

Prescription of medication may be seen as a doctor's responsibility but we need to ensure our MDT staff has up to date knowledge of types of medications used, their indications, contra-indications, monitoring requirements, and side effects.

Regular teaching, online modules and experience of shadowing with doctor's clinics should be offered to all the MDT staff.

